# OTA-Grapes: A Mechanistic Model to Predict Ochratoxin A Risk in Grapes, a Step beyond the Systems Approach

**DOI:** 10.3390/toxins7083012

**Published:** 2015-08-06

**Authors:** Paola Battilani, Marco Camardo Leggieri

**Affiliations:** Department of Sustainable Crop Production, Università Cattolica del Sacro Cuore, Via Emilia Parmense 84, 29122 Piacenza, Italy; E-Mail: marco.camardoleggieri@unicatt.it

**Keywords:** mycotoxin, fungi, climate, infection cycle, temperature, water activity, rain, *Aspergillus carbonarius*

## Abstract

Ochratoxin A (OTA) is a fungal metabolite dangerous for human and animal health due to its nephrotoxic, immunotoxic, mutagenic, teratogenic and carcinogenic effects, classified by the International Agency for Research on Cancer in group 2B, possible human carcinogen. This toxin has been stated as a wine contaminant since 1996. The aim of this study was to develop a conceptual model for the dynamic simulation of the *A. carbonarius* life cycle in grapes along the growing season, including OTA production in berries. Functions describing the role of weather parameters in each step of the infection cycle were developed and organized in a prototype model called OTA-grapes. Modelling the influence of temperature on OTA production, it emerged that fungal strains can be shared in two different clusters, based on the dynamic of OTA production and according to the optimal temperature. Therefore, two functions were developed, and based on statistical data analysis, it was assumed that the two types of strains contribute equally to the population. Model validation was not possible because of poor OTA contamination data, but relevant differences in OTA-I, the output index of the model, were noticed between low and high risk areas. To our knowledge, this is the first attempt to assess/model *A. carbonarius* in order to predict the risk of OTA contamination in grapes.

## 1. Introduction

Grapes and their derivatives (grape juice, raisin and wine) are important foodstuff for the human diet, while wine is considered an income commodity [1]. Several diseases can affect grapes during the growing season, causing quantitative and qualitative yield losses, but the menace to human health is actually only associated with fungi belonging to *Aspergillus* section *Nigri* (commonly called black *aspergilli*) and their possible ochratoxin A (OTA) production [2]. These fungi colonize grape berries from setting, with increasing incidence moving towards ripening. Among the species isolated in grapes, those belonging to *A. niger* aggregate (*i.e.*, *A. niger* and *A. tubingensis*) dominate [3,4], but *A. carbonarius* is confirmed as the key one responsible for OTA contamination. In fact, almost 100% of the strains of this fungus are strong OTA producers [5,6], in contrast with 5%–10% reported for the *A. niger* aggregate; besides, they produce lower amounts of the toxin compared to *A. carbonarius* [7,8]. 

Ochratoxin A is a fungal metabolite dangerous for human and animal health due to its nephrotoxic, immunotoxic, mutagenic, teratogenic and carcinogenic effects [9,10,11]. It is confirmed to accumulate in kidney [12,13], considered to play a major role in Balkan endemic nephropathy [14,15] and classified by the International Agency for Research on Cancer [16] in group 2B, possible human carcinogen. This toxin has been stated as a wine contaminant since 1996 [17] and confirmed by several surveys after this first report [3]. The range of OTA content in wine produced in Europe from 1996–2008 varied between 0.01 and 3.4 µg/L; the contamination level differed between years and geographic areas of grape origin, with a north-south positive gradient in Europe [3]. Environmental conditions are crucial for black aspergilli growth and OTA production [18], mainly air temperature, rainfall [8] and relative humidity [19]. *Aspergillus carbonarius* grows optimally at 30–35 °C, and the optimal water activity (*a*_w_) reported varied from 0.93–0.99 with the widest range at 25–30 °C. Regarding OTA production, optimum conditions were stated at 0.95–0.98 *a*_w_ and 15–20 °C or 30–35 °C, depending on the strains, but irrespective of their geographical origin [3,20,21,22,23].

The occurrence of OTA in wine may be mitigated by adopting appropriate management strategies in the vineyard [24,25,26]. The cultural practices considered relevant regard: (i) pest control, mainly *Lobesia botrana* [27,28,29,30]; (ii) disease control, *i.e.*, powdery mildew (*Erysiphe necator*, *Oidium tuckeri*) [31,32,33]; and (iii) chemical and biological control actions towards black *Aspergilli* [32,34,35]. No clear evidence resulted regarding the role of fertilization/irrigation, grape variety and trellising system [24,30,36,37]; they play a role, but a quantitative effect is quite difficult to demonstrate due to their strong inter-relationship [38]. 

Modelling the complex patho-system *A. carbonarius*-grapes-environment, along the crop growing season, could enable the prediction of ongoing risk of OTA contamination until harvest. This crucial information can support the optimization of grape management, pre- and post-harvest, and the mitigation of OTA content in grapes.

The aim of this study was to develop a conceptual model for the dynamic simulation of the *A. carbonarius* life cycle in grapes along the growing season, OTA production in berries included, following the principles of “system analysis”. Functions describing the role of weather parameters in each step of the infection cycle were developed and organized into a prototype model. 

## 2. Results 

### 2.1. Prototype Predictive Model OTA-Grapes 

**Figure 1:**
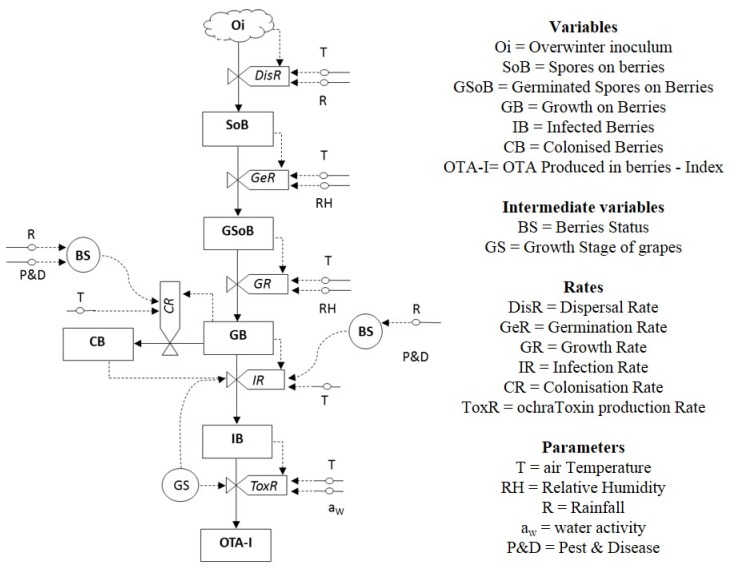
Relational diagram of OTA-grapes (OTA, ochratoxin A) and the predictive model for *Aspergillus carbonarius* growth and ochratoxin A (OTA) production in grapes.

The relational diagram, slightly modified with respect to the first published version [3], is shown in Figure 1. The inoculum source, mainly mycelium overwintering in the soil [39], not quantified in the model (Oi), is the opening state variable; spores produced from this source are air-borne to bunches. The amount of spores disseminated to berries (SoB) is regulated by a dispersal rate (DisR), depending on the temperature (T) and rain (R) regimes. The germination rate (GeR) regulates the amount of spores that can germinate on berries (GSoB), and the growth rate (GR) regulates the growth of germinated spores (GB). Then, the mycelium can follow two infection pathways: it can (i) stay on berries and colonize their skin (CB); or (ii) penetrate and infect berry flesh (IB). The pathway depends on the berries’ status (BS), mainly (i) presence/absence of openings in the skin and (ii) grape growth stage [37]. Many factors can determine skin damages, but R during ripening and pest-disease (P&D) presence play a major role. CB and IB are considered for the OTA production index (OTA-I) through a toxin production rate (ToxR). The influence of grape variety has been removed in this version of the relation diagram because of the lack of useful data to develop a proper function; this information is now accounted for by GS.

### 2.2. Overwintering Inoculum (Oi)

Spore production depends on the amount of fungal inoculum in soil and ecological conditions. The inoculum was assumed to be naturally available, based on many surveys managed in different vine growing areas signaling black aspergilli on berries at setting [40,41,42]. 

Sporulation of *A. carbonarius* was reported to take place with a combination of *T* and relative humidity (RH) equal or higher than 25 °C and 85%, respectively, referring to quantitative data collected for *A. niger* sporulation on onion [43,44]. No quantitative data are available in the literature regarding the sporulation of black aspergilli on grapes in different ecological conditions, but based on the experience with the AFLA-maize model, developed to predict the behavior of *A. flavus* in maize [45], we assumed that spores are available with a combination of *T* and RH equal to or higher than 10 °C and 80%, respectively. This was included as a binomial variable (yes/no answer) in the model.

### 2.3. Spore Dispersal (DisR)

Dispersal of *A. carbonarius* spores was confirmed to be wind borne by Kazi, Emmett, Nancarow and Clarke [39], but they did not publish quantitative data. Therefore, spore dispersal was included as a binomial variable, too, and it was assumed as not possible on rainy days or with RH ≥ 80%, similarly to the statement used in the AFLA-maize model for *A. flavus* [45].

**Table 1:** Summary of the functions used to fit data and to obtain rate variables for each step of the infection cycle of *A. carbonarius* relevant for modelling and the parameters computed.

Rate	Variable	Function	Ref. text	Parameters *							
				*a*	*b*	*c*	*x*	β	*R*^2^	*T*_min_	*T*_max_
*GeR* ^†^	*T*	Bete	1	4.879	0.023	9.921	- ^#^	-	0.98	5	45
	*a_w_*	Polynomial		−0.458	89.842	−4323.4	-	-	1	-	-
*GR*	*T*	Bete	1	4.692	1.277	1.899	-	-	0.84	5	45
	*a_w_*	Linear		-	-	-	7.275	−6.257	0.83	-	-
*ToxR*	*T*	Bete	1	2.778	0.650	2.760	-	-	0.54	10	37
	*T_1_*	Bete	1	2.770	0.562	6.559	-	-	0.87	10	37
	*T_2_*	Bete	1	9.915	3.201	0.588	-	-	0.76	10	37
	*a_w_*	Linear		-	-	-	8.153	−7.135	1	-	-

^#^ Not applicable. ^†^ Data published by Camardo Leggieri, Mitchell, Aldred, Battilani and Magan [46]. * *a*, *b* and *c* are the parameters of the Bete equation; x and β are the parameters of linear regression; *R*^2^ is the coefficient of determination; *T*_min_ and *T*_max_ are the cardinal *T* values.

### 2.4. Germination Rate (GeR)

Spore germination is mainly influenced by *T* and *a*_w_. Information available in the literature about this step of the infection cycle [47,48] till recently was poor, but suitable quantitative data on *A. carbonarius* germination on grape flesh and skin and their modelling were recently published [46]. Briefly, conidia germination occurred more rapidly on grape flesh (6 h), followed by grape skin (24 h), under the optimal conditions of 30–35°C and 100% RH. The combined effect of *T* and RH/*a*_w_ on the germination of *A. carbonarius* conidia in grape berries (both on skin and flesh) was modelled with a Bete equation (*T*) and a polynomial equation (*a*_w_); these functions were used for GeR in the OTA-grapes model developed here (Table 1).

### 2.5. Growth Rate (GR)

The GR of *A. carbonarius* depends on *T* and *a*_w_.

**Figure 2:**
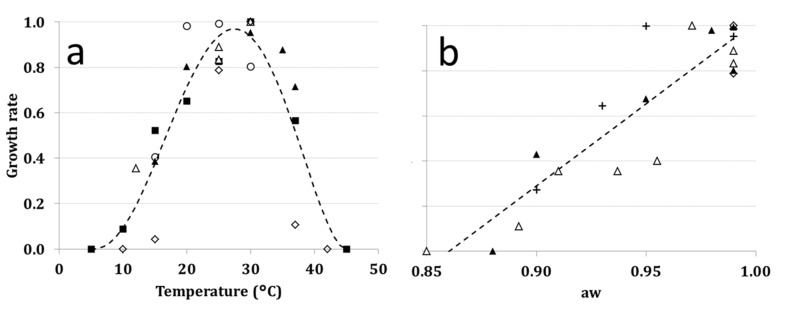
Growth rate of *Aspergillus carbonarius* at different regimes of (**a**) temperature (T; 5–40 °C) and (**b**) water activity (*a*_w_; 0.85–0.99). The symbol reference is reported in Table 1.

Based on quantitative data published by several authors, [8,49,50,51,52] (Table 2, Figure 2a), GR was computed as a function of *T* using Bete Equation (1) [53].

(1) $$GR\left(T\right)={\left(a\cdot {\left(Teq\right)}^{b}\cdot \left(1-Teq\right)\right)}^{c}$$

where *Teq* is the equivalent of *T*, computed as:

(2) Teq=(T−Tmin)(Tmax−Tmin)

*T*_min_ and *T*_max_, the cardinal temperatures, are 5 °C and 45 °C, respectively, in this case. 

Estimated parameters are provided in Table 1.

Regarding *T* (Figure 2a), *Aspergillus carbonarius* growth starts from 10 °C; GR increases up to 25–30 °C (optimal conditions), decreasing at higher *T* values; growth was never observed at 45 °C. 

Regarding *a*_w_ (Figure 2b), the best fit of data was obtained with a linear equation. Water activity = 0.88 is the lowest limit for growth, while *a*_w_ = 0.98–0.99 is the optimal condition.

**Table 2:** Symbols included in Figure 2 and Figure 3 for the references, including quantitative data used in this study to run regression analysis and implement equation parameters for growth rate (GR) and ochratoxin production rate (ToxR) reported in Table 1.

Symbols	Selected paper	GR	ToxR
∆	Astoreca *et al.* [49]	*	
■	Battilani, Giorni and Pietri [8]	*	
▲	Belli *et al.* [50]	*	
+	Belli *et al.* [54]		*
□	Esteban *et al.* [55]		*
●	Lasram, Oueslati, Valero, Marin, Ghorbel and Sanchis [21]		*
◊	Leong *et al.* [51]	*	*
♦	Romero, Pinto, Patriarca and Vaamonde [22]		*
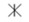	Selouane, Bouya, Lebrihi, Decock and Bouseta [23]		*
○	Spadaro *et al.* [52]	*	

* The papers that contributed to developing the functions for growth rate (GR) and ochratoxin production rate (ToxR).

### 2.6. Infection Rate (IR) 

Fungal infection strictly depends on berry status. The infective capacity of eight strains of *A. carbonarius* on damaged/undamaged grape berries was considered by Belli *et al.* [56]. Damaged berries resulted in being more susceptible to the infection compared to intact ones. Relative humidity ≤ 80% resulted in being not conducive for infection; in fact, with RH = 80%, 25% of the studied strains did not infect damaged berries. On un-damaged berries, no infection occurred with RH between 75% and 80%. 

Conducive conditions for the infection are computed by IR, taking into account berries status (with/without lesions) and grape growth stage. In fact, *A. carbonarius* infection is promoted when: (i) berries have lesions; (ii) they are close to harvest (late ripening); and (iii) air temperature is around 30 °C and RH ≥ 85% [37,56]. 

### 2.7. Toxin Production Rate (ToxR)

Ochratoxin A production at different *T* regimes has been studied by several authors [21,22,23,53,54,55] (Table 2). 

**Figure 3:**
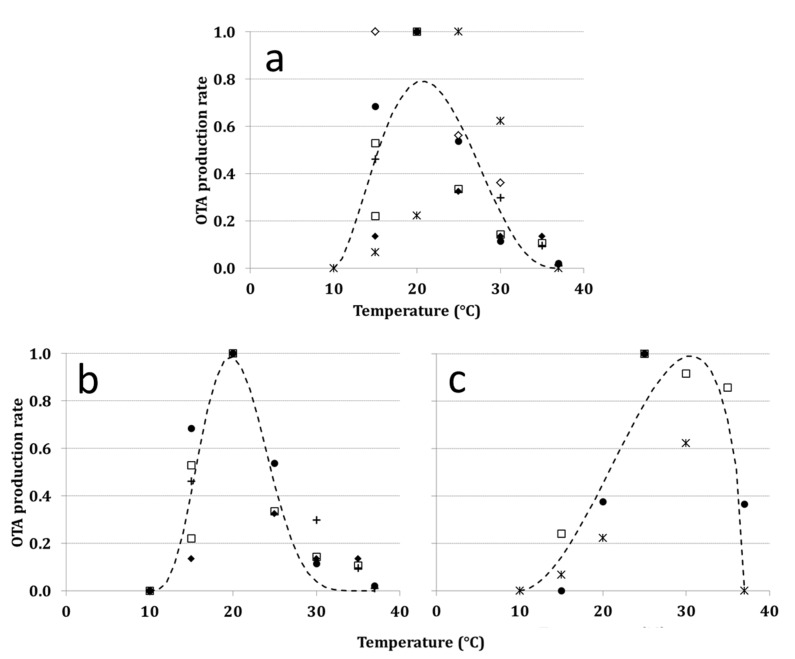
OTA production rate (ToxR) for *A. carbonarius* considering: (**a**) all available data; (**b**,**c**) data shared based on the dynamic of OTA production and according to the optimal temperature of different strains (20 and 25 °C, respectively). Symbols refer to data from the literature, and the references are reported in Table 2.

The Bete equation was chosen to fit all data as a function of *T*, to compute ToxR_T_, but it resulted in a poor data fitting (*R*^2^ = 0.54; Table 1, Figure 3a). Strains with significant difference in optimal *T* were clearly distinguishable in published papers; therefore, data were shared in two groups based on fungal strain behaviors in different *T* regimes. The Bete equation was run for each of the two derived datasets, and *R*^2^ improved significantly for both functions developed (*R*^2^ > 0.75; Table 1, Figure 3b–c).

Ochratoxin A production rate in different *a*_w_ regimes (ToxR_aw_) was successfully fitted by a linear function (Table 1). No OTA production was described at *a*_w_ regimes ≤ 0.87 and ToxR_aw_ increased from 0.90–0.99, the optimum *a*_w_ for OTA production. 

### 2.8. Effect of Weather Data on ToxR_T_

In the fungal population naturally present in vineyards, the prevalence of strains belonging to the two groups previously described for toxin production rate (ToxR_T1_ and ToxR_T2_) is unknown. Then, data from 48 weather stations (see Materials and Methods for details) were used to compute ToxR_T_, for the two strain types, giving them a different role. The two functions developed (ToxR_T1_ and ToxR_T2_) were considered to complementarily contribute between 0% and 100% (100% as the global contribution; Table 3). The overall mean for ToxR_T1_ and ToxR_T2_ values (output dataset, computed including 48 weather stations and 11 combinations of the two strain types) was 638 and the range of variation was limited, both between years and areas of weather data collection, with a minimum and maximum in 2010 (±0.2–±3.1) and in 2011 (±15.8–±27.6), respectively.

One-way ANOVA was applied to the output dataset. Significant differences (*p* ≤ 0.01) were noticed regarding ToxR_T_ intra-growing areas and intra-years (*p* ≤ 0.05) (Table 3). The index obtained with equal contribution of both strain type (50%/50%) was significantly different only from the 0%/100% and 100%/0% combinations. Therefore, it was assumed as a correct approach to give the same role in the predictive model to the two population types, therefore to the two functions, it being considered impossible to have only one strain type in a grapevine growing area.

**Table 3:** Mean values of ochratoxin A production rate (ToxR_T_) obtained running the two functions’, ToxR_T1_ and ToxR_T2_ (see Table 1), generated clustering data, based on fungal strain behavior in different *T* regimes, in different complementary proportions ranging from 0%–100%.

% ToxR_T1_	% ToxR_T2_	Mean ToxR_T_ *
0	100	603.24 a
10	90	611.84 ab
20	80	620.44 ab
30	70	629.05 ab
40	60	637.65 ab
50	50	646.25 b
60	40	654.86 bc
70	30	663.46 bc
80	20	672.06 bc
90	10	680.66 bc
100	0	689.27 c

* Different letters means significant differences (*p* ≤ 0.01, obtained with the Tuckey test).

### 2.9. Model Output (OTA-I)

OTA-grapes was run using as input meteorological data collected in years/georeferenced locations where OTA contamination in grapes was quantified at harvest in previous research studies. In particular, two meteorological stations from the Apulia region, Southern Italy, in 1999 and 2000 [8], and two in the Emilia Romagna region, Northern Italy, in 2012–2013 were selected (Battilani *et al.* [57]). OTA above the EU legal limit was detected in 1999 in Apulia, while it was absent in 2000 [8]. OTA was never detected in Emilia Romagna. OTA-I was high in Apulia, in both years, higher in 1999 compared to 2000, 3819 *versus* 2942, while very low levels were obtained running OTA-grapes in Emilia Romagna, six and eight respectively in 2012 and 2013. 

## 3. Discussion

Ochratoxin A occurrence in grapes and wine has been largely reported after 1996 in several European countries [3]. Surveys agree on the decreasing gradient of OTA incidence and severity from red, rosè and white wines [8,58,59,60]. Higher contamination is reported for dessert wines, due to the over-maturation/drying of grapes before the wine making process, in agreement with high contamination reported in raisin and currant. In general, grape derivatives produced in South Europe and North Africa are more severely affected by OTA than those from the temperate regions of the middle of Europe [17,61,62].

The proportion of wine in which OTA is detected is high (>50%) in some countries, but the legal limit of 2 µg/L fixed by the EC [63] is rarely exceeded [3]. Therefore, OTA contamination seems a problem actually under control, both in conventional and organic farming, in Europe, as confirmed by Comuzzo *et al.* [64], who found OTA below the legal limit in 95% of the 971 wine samples analyzed. 

The alarm regarding OTA contamination in wine, launched around the end of the 20th century, is now apparently back, thanks to cropping system actions enforced to improve grapevine wellness, mainly preventing pest and disease damages on berry, including the control of black aspergilli, the optimization of postharvest logistic and of wine making [3,18,29,31,65]. Nevertheless, this is true for well-studied and well-managed grape growing areas, but the modelling approach can also support poorly-studied areas, if meteorological data are available, and this can help in grapevine planning and management, more useful with the extreme climatic events frequently faced in recent years. Global warming has markedly shifted the distribution of temperature variability and extremes and the precipitation patterns with significantly higher frequency of exceptionally unfavorable years for several crops [66]. Therefore, it is very important to organize a predictive system for OTA risk in grapes in order to support all of the operators in the grape chain, including policy makers and risk managers, in their strategic and tactic decisions, especially in climate change scenarios, as recently stressed by Battilani and Camardo Leggieri [67] for aflatoxin in maize.

*Aspergillus carbonarius* is the key fungus for OTA production in grapes; it is commonly present in vineyards [58,68,69], but relevant OTA contamination is quite rare [3]. Therefore, OTA-grapes, the predictive model developed in this study, focused on *A. carbonarius* to support the prediction of OTA risk in year/area combinations. To our knowledge, this is the first attempt to assess/model the role of *A. carbonarius* strains in order to optimally represent what happens in reality, which is the principal aim of predictive modelling [70,71]. Empirical models were developed to describe the influence of *T*, *a*_w_ and growing medium on specific steps of the infection cycle [21,46,72,73,74,75,76]. They were a very good support for our modelling; suitable equations were developed to describe rate variables, with *R*^2^ ≥ 0.83 in all of the equations included in OTA-grapes.

In our study, working on the OTA production rate as a function of T, it emerged that fungal strains can be shared in two different clusters, based on the dynamics and the optimum of OTA production as a function of T. This is in agreement with several molecular studies that underlined the great biodiversity of *A. carbonarius* strains, often unrelated to isolation area and harvest year [21,22,77,78]. The OTA production rate could be considered a potential weakness of the model; in fact, realizing the relevant diversity between strains in OTA production dynamic and it being impossible to know the composition of natural *A. carbonarius* populations, the statistical approach was used, and an equal contribution of the two strain clusters to OTA synthesis was assumed. Besides, quantitative data available were all obtained *in vitro* on artificial media, and this could face differences when berries are considered as the growing medium, an issue stressed also for AFLA-maize [45]. Nevertheless, we consider the approach appropriate, the interest being focused on predicting the level of risk, not the amount of OTA content in berries, in agreement with the common opinion that the latter is almost impossible to predict. 

The reduction of *A. carbonarius* population in vineyards could be successfully achieved by control measures, which focus on the application of chemical/biological fungicides [65,79]. Chemical formulates, such as Azoxystrobin, Dinocap, Chorus and Switch, have been tested for their efficacy for preventing OTA accumulation in grapes with successful results [34,79]. In view of avoiding the use of fungicides when unnecessary and to face integrated pest management (IPM), actually mandatory in Europe [80], OTA-grapes increased the patho-system understanding [81], and it could be a good support for stakeholders in the grape chain being able to give a daily prediction on the level of risk in different areas and years. 

Model validation, the final mandatory step to release reliable predictions, can be generally summarized as the comparison between model predictions and real observations of a certain phenomenon, e.g., disease outbreak, disease incidence or OTA contamination in grapes, in this case to check their agreement. Model validation requires a consistent set of field data on the modelled phenomenon, possibly shared above and below a reference threshold, the legal limit in this case. Unfortunately, most of the available data reports OTA contamination below the legal limit; then, the definition of a probability function for OTA contamination was not possible. In our study, we observed a certain level of agreement between OTA-grapes predictions/output (OTA-I) and OTA level detected in the field, for the vineyards considered. In fact, as reported in the Results section, OTA-I was greater for OTA-contaminated grapes. This is an encouraging result, even if not yet supported by a consistent validation. 

In conclusion, OTA-grapes, even if not yet validated, represents a step beyond the systems approach. Predictive mycology is useful to assess fungal growth and, therefore, mycotoxin contamination in food products [82], and it should receive more attention to improve food safety [75,83]. It must be kept in mind that model predictions, even if reliable, will never be 100% correct. Therefore, supporting tools, like rapid diagnostics applied at harvest [84], paired with risk prediction, should make model support to farmers more robust [67].

## 4. Material and Methods

This study followed the approach previously described for AFLA-maize, a predictive model for *Aspergillus flavus* in maize, another important mycotoxin-producing fungus with a wide range of potential hosts [45]. All quantitative data available in the literature regarding the *A. carbonarius*-grape patho-system were considered. 

### 4.1. Literature Search 

Following the protocol developed by Okoli and Schabram [85], published papers that contain quantitative data for the patho-system of interest were retrieved. The papers had to comply with the following criteria, to be used for model development: (i) containing original data on the ecology or epidemiology of *A. carbonarius* (preferably, but not exclusively, the interaction with the host crop); and (ii) be published in peer-reviewed journals or proceedings of top level congresses. The literature search was carried out in the CAB Abstract database (http://www.cabdirect.org). All papers were reviewed, and in case they met the eligibility criteria, quantitative data reported were collected in a proper database.

### 4.2. Model Development

The relational diagram developed by Battilani and Silva [3] was considered, slightly modified, to develop the prototype predictive model named OTA-grapes (Figure 1). 

Briefly, in the relational diagram [86], the status of the fungus at a certain time is represented by boxes, intended as state variables (Oi, SoB, GSoB, GB, CB, IB and OTA-I); clouds are used when it is not possible to quantify the state variable, commonly the overwintering inoculum (*i.e.*, Oi, Figure 1). The flow from one state to the following is driven by constants/parameters (T, R, RH, *a*_w_, P&D) or intermediate variables (BS and GS), essentially derived from weather or crop data. Rate variables are represented by “valves” (DisR, GeR, GR, IB, CR, ToxR). They are described by mathematical functions, and the output is reported on a 0–1 scale, with 1 intended as the rate occurring at optimal conditions; the flow to the next stage is stopped due to limiting conditions when the rate is 0.

The linear or the non-linear regression procedure of the statistical package PASW statistics (Ver. 21, 2012) was used to develop mathematical functions to be used for all rate variables included in the relational diagram. Functions were finally linked in a coherent mathematical framework to calculate the output, *i.e.*, the OTA cumulative index (OTA-I).

### 4.3. Grape Growth Stage Model 

The intermediate variable GS (= crop growth stage) is a crucial component of the predictive model. A sub-model able to predict grape growth stages based on normal heat hours (NHH) summation was considered; it was developed for several grape varieties by Mariani *et al.* [87] and validated in Italy by Cola *et al.* [88] with good results. NHH are hours weighted (in the scale 0–1) on plant activity *T* range (*T*_min_ and *T*_max_) and optimal *T* (*T*_opt_). 

The growth stages considered in OTA-grapes were BBCH 71, 75, 81, 85 and 89 [89], intended respectively, as fruit set, berries pea-sized, beginning of ripening/veraison, softening of berries and berry ripe (harvest time).

### 4.4. OTA-Grapes Data Input and Output

Hourly data on air temperature (*T*, °C) and relative humidity (*RH*, %) and rain (*R*, mm) are the mandatory data input. Data from 1 January are requested for crop phenology modelling; when early veraison is reached, the *A. carbonarius* predictive model starts. Model running continues till berries are ripe, computing OTA-I on a daily base; it accumulates in time, and this accumulated value is the output of the OTA-grapes predictive model. 

### 4.5. Meteorological Data

Hourly data on T, RH and R along grape growing season, available from previous studies, were considered. Eight meteorological stations placed in the Emilia Romagna region (Northern Italy) in 6 years 2006–2011 were used to test the effect of weather data on ToxR_T,T1,T2_. 

Two stations from the Apulia region, Southern Italy, in 1999 and 2000 [8], and 2 in the Emilia Romagna region, Northern Italy, in 2012–2013 were selected due to the availability of data on OTA contamination in grapevines grown close to the stations. They were used to validate the OTA-grapes model. 

### 4.6. Effect of Weather Data on ToxR_T_

Two functions were developed for the rate variable ToxR_T_ in different *T* regimes to describe 2 groups of *A. carbonarius* strains based on their behavior in different *T* regimes (ToxR_T1_ and ToxR_T2_ for optimal *T* at 20 and 25 °C, respectively). The 2 functions were run for all 48 weather stations selected in Emilia Romagna in the period 1 August–15 September, approximately between early veraison and ripening for grapes in the growing area considered, for all 6 years. 

The output values obtained for the 2 functions, in each weather station and year, were combined giving to each ToxR_T_ a contribution changing from 0%–100%, with a step of 10%. The aim was to simulate a different role of the 2 types of strains. Eleven ToxR_T_ values were therefore obtained for each station and year, 528 values in total. One-way ANOVA was run with these data using alternatively year and weather station as factor and replicate; the Tukey test was applied to highlight significant differences between means. 

### 4.7. Preliminary OTA-Grapes Validation

The model was validated using data from 4 natural epidemics recorded in two grape-growing regions of Italy in four years (1999, 2000, 2012, 2013). Two vineyards were monitored in Apulia in 1999 and 2000, and they included the only data available from previous research activities, regarding OTA contamination in Italy above the EU legal limit. Forty vineyards were sampled in Emilia Romagna in 2012 and 2013; OTA was never detected in these vineyards.
